# Vascular endothelial growth factor genetic polymorphisms and susceptibility to age-related macular degeneration in Tunisian population

**DOI:** 10.1186/2050-7771-2-15

**Published:** 2014-08-18

**Authors:** Imen Habibi, Imen Sfar, Ahmed Chebil, Fedra Kort, Rim Bouraoui, Salwa Jendoubi-Ayed, Mouna Makhlouf, Taïeb Ben Abdallah, Leila El Matri, Yousr Gorgi

**Affiliations:** 1 Research Laboratory of renal Transplantation and Immunopathology (LR03SP01), University Tunis El Manar, Immunology Laboratory, Charles Nicolle Hospital, Charles Nicolle Hospital, Bd 9 Avril, Tunis 1006, Tunisia; 2 Oculogenetic Research Unit, Department B of Ophtalmology, Hedi Rais Institute of Ophthalmology, Tunis, Tunisia

**Keywords:** AMD, Macula, VEGF

## Abstract

**Purpose:**

Three VEGF SNPs (−2578) C/A, (+405) G/C and (+936) C/T were investigated in Tunisian exudative AMD patients in order to determine their association with the disease susceptibility and their influence to intravitreal bevacizumab therapy response.

**Methods:**

145 AMD patients and 207 age-matched controls were included. 68 patients were treated with intravitreal bevacizumab. SNPs genotyping were performed using direct sequencing. The serum VEGF was assayed by ELISA (R&D).

**Results:**

The (+405) CC and (+936) TT genotypes were higher in AMD patients than in controls (*p* = 5 × 10^−6^ and *p* = 0.021, respectively). The mean plasma levels of VEGF were statistically higher in AMD patients (84.22 pg/ml) than in controls (15 pg/ml). Three months after bevacizumab treatment, 52 patients (85.6%) were classified as good responders (GR) and 16 (14.4%) as poor responders (PR). The mean plasmatic-VEGF levels in GR patients was higher (86.61 ± 80.30 pg/ml) than in PR patients (47.12 ± 45.74 pg/ml) *(p = 0.086)*. The patients with genotype homozygous TT (+936) would be PR compared to those carrying CT and CC genotypes. Whereas, those with AA (−2578) genotype would be GR compared with others genotypes *(p* = 0.014; *p* = 0.042 respectively).

**Conclusions:**

Our results show that VEGF genetic variants may contribute to the susceptibility to neovascular AMD in Tunisian patients.

## Introduction

Age-related macular degeneration (AMD) is a progressive chronic inflammation that affects the cells of the retinal pigment epithelium (RPE) and promotes the formation and deposition of phospholipid material retinal drusen. This maculopathy progresses to degeneration in two forms: “wet AMD” and “Dry AMD”. Indeed, most visual loss occurs in the late stages of the disease due to one of two processes: choroïdal neovascularization (CNV) and geographic atrophy [1,2]. Etiological research suggests that AMD is a complex disease, caused by the interactions of several genetic and environmental factors. Different evidences have supported that in exudative AMD form, inflammatory lesions are associated with a high expression and synthesis of growth factors such as vascular endothelial growth factor (VEGF) which is a key regulatory factor in angiogenesis and vascular permeability in both physiological and pathological states [3]. VEGF expression has been shown in experimental choroidal neovascularization and shown to induce CNV growth in animal models [4]. Therefore, it would be involved in the development of CNV in this disease.

More recently, attention has been focused on the function of VEGF in light of its role as a therapeutic target and VEGF-inhibitors have been used in successful therapy of exudative AMD [5,6]. The most important advance in the treatment of neovascular AMD is the development of anti-vascular endothelial growth factor (anti-VEGF) therapeutic agents that preserve and improve visual acuity by arresting choroidal neovascular growth and reducing vascular permeability. Thus, VEGF is a potential candidate for genetically influencing AMD susceptibility, based on its functional relevance to AMD pathophysiology.

VEGF gene exhibits many single nucleotide polymorphisms (SNPs) that might influence qualitatively and/or quantitatively its expression. Previous studies have shown that the genetic variations in the VEGF gene influence the rate of the VEGF protein synthesis [7-9]. The human VEGF-A gene is located on chromosome 6 (6p21.3) and is organized into eight exons [10]. Several different isoforms of VEGF are generated by alternate splicing of the VEGF-A gene [11]. Of these, the VEGF_165_ isoform (named according to the number of amino acids), is the most abundant and corresponds to a 23 kDa polypeptide, constituting a monomer of homodimeric human VEGF-A the role of vascular endothelial growth factor (VEGF) in inflammatory bowel disease [12].

In this context, three common VEGF SNPs (C-2578A, rs699947), (G + 405C, rs2010963) and (C + 936 T, rs3025039), in the promoter, the 5′Untranslate and the 3′Untranslated regions respectively, were investigated in Tunisian exudative AMD patients in order to determine their association with the disease susceptibility, their influence to the level of production of this glycoprotein and the response to intravitreal bevacizumab therapy.

## Material and methods

### Patients

One hundred and forty five unrelated Tunisian patients with exudative AMD were collected from the Department B of Ophthalmology, Hedi Rais Institute of Ophthalmology, Tunis, Tunisia.

The diagnosis of AMD was established on the basis of clinical examination, fundus photographs and fluorescein angiography results. Fundus findings in each eye were classified based on a standardized set of diagnostic criteria established by the International Age-Related Maculopathy Epidemiologic Study [13]. Data obtained from each patient at the ophthalmology institute included age, gender, personal/familial history of AMD and risk factors (tobacco use, arterial hypertension, hypercholesterolemia and cardiovascular risk). Clinical and epidemiological characteristics of patients are summarized in Table 1.

**Table 1:** Clinical and epidemiological characteristics of patients

	**Patients (n = 145)**	**G1(n = 117)**	**G2 (n = 28)**
Sex ratio M/W	99/46	83/34	16/12
Mean age (years ± SD)	73.14 ± 8.06	73.22 ± 8.09	72.71 ± 8.77
Range (years)	(52–92)	(52–92)	(53–89)
***Exudative AMD subtypes:*** *n (%)*			
▪ PC CNV	40(27.59)	40(34.18)	-
▪ O CNV	53(36.55)	53(45.29)	-
▪ MC CNV	24(16.55)	24(20.51)	-
▪ Fibrovascular scarring	28(19,31)	-	28(100)
***Risk Factors:*** *n (%)*			
▪ Smoker	91(62.75)	72(61.53)	19(67.85)
▪ Arterial Hypertension	54(37.24)	46(39.31)	8(28.57)
▪ Hypercholestérolemia	28(20.68)	22(18.80)	6(21.42)
▪ Heart risk	27(18.62)	21(17.94)	6(21.42)
▪ History of AMD	9(6.20)	8(6.83)	1(3.57)
▪ Cataract surgery	22(15.17)	17(14.52)	5(17.85)

M: men; W: women; n: number; SD: standard deviation; CNV: choroidal neovascular.

PC: predominantly classic; O: occult; MC: minimally classic.

Exudative AMD patients were classified in two groups. The first group (G1) composed by 117 patients with active neovascular form and divided into three subtypes including 40 (34,2%) predominantly classic cases without occult CNV, 53 (45,3%) occult CNV and 24 (20,5%) minimally classic CNV, according to the guidelines from the international classification. The second group (G2) included 28 patients with cicatritial lesion form.

According to the standard protocol for anti-VEGF therapy, 68 of G1 patients (58.11%) were treated with 3 initial monthly intravitreal bevacizumab injections (1.25 mg in 0.05 ml). After this therapy, improving visual acuity (VA), as a 2-lines gain (a margin of 10 ETDRS lettres) was used to compare response to treatment. Patients were classified into:

–Good responders (GR): defined as patients with a gain of ≥ 10 ETDRS letters and those who demonstrated stability in visual acuity (a gain or loss of < 5 lettres) after the 3 bevacizumab injections.

–Poor responders (PR): defined as patients with a loss of > 10 ETDRS letters in post-therapy.

#### Controls

Two hundred and seven subjects blood donors older than 50 years and having undergone a complete ophthalmological examination with a normal fundus test, served as control group.

All patients and controls were fully informed of the purpose and procedures of the study, and informed consent was obtained from all patients before they were enrolled in the study.

#### Methods

Venous blood of patients and controls was collected in EDTA tubes. Plasma was frozen at −20°C for determination of VEGF plasma levels and genomic DNA was extracted for molecular study using a salting-out procedure [14].

### Determination of VEGF plasma levels

Plasmatic VEGF levels of 104 patients included in this study were measured using the Human VEGF Quantikine ELISA kit (R&D Systems, Inc., Minneapolis, MN, USA), according to the manufacturer recommended protocol. VEGF levels in patient samples were expressed in pg/ml. The normal mean value of VEGF is 15 ± 4.5 pg/ml. In 68 G1patients, this assay occurred before treatment.

### Genotyping of VEGF gene polymorphisms

In the promoter region, the VEGF (−2578) C/A gene polymorphism was determined using the previously described PCR restriction fragment length polymorphism (RFLP) method. Primers used were as follows15:

Forward 2578 sens:

5′-ATAAGGGCCTTAGGACACCA-3′

Reverse 2578 Antis:

5′-GCTACTTCTCCAGGCTCACA-3′

PCR was carried out in a final volume of 20 μl containing 100 ng of genomic DNA, 1.5 mM MgCl_2_, 0.2 mM dNTP, 10 pmol of each primer and 0.5U of Taq DNA polymerase (Promega, USA). The PCR product were digested for 2 hours at 37°C with restriction endonuclease *BglII* (SIBENZYME) at a final concentration of 4 units, fragments were analyzed on 3% agarose gels stained with éthidium bromide. The A allele remained uncut, while the C allele was cut into two fragments of 212 and 264 bp.

The VEGF (+405) C/G in the 5′Untranslated region and (+936) C/T in 3′Untranslated region gene genotyping were performed by a polymerase chain reaction (PCR) in a final volume of 20 μl containing 5 pmol of each primer: 5′-ATTTATTTTTGCTTGCCATT-3′ (forward primer +405S) and 5′-GTCTGTCTGTCTGTCCGTCA-3′ (reverse primer +405AS); 5′-AAGGAAGAGGAGACTCTGCGC-3′ (forward primer +936S) and 5′-TATGTGGGTGGGTGTGTCTACAGG-3′ (reverse primer +936AS) respectively, 50 ng of genomic DNA, 1.5 mM of MgCl_2_, 0.2 mM dNTP, 10 pmol of each primer and 0.5U of Taq DNA polymerase (Promega, Madison, WI, USA).

The DNA was denatured at 94°C for 4 minutes, prior to 30 cycles of amplification. The conditions used for each cycle were denaturation for 30 seconds at 94°C, annealing for 30 seconds at 60°C, and extension for 1 minute at 72°C. The 30 amplification cycles were followed by a final extension step at 72°C for 5 minutes. The PCR products were resolved in 2% agarose gels stained with ethidium bromide. The amplified fragments were then sequenced in forward direction using the forwards primer in an ABI PRISM Dye Terminator Cycle Ready Reaction kit (Applied Biosystems) under recommended conditions. Sequenced samples were purified using Centri-Sep columns (Dye EXTm 2.0 Spin Kit, Qiagen) according to manufacturer’s instructions, loaded in a PE ABI Prisms 310 Genetic Analyzer (Perkin Elmer) and analyzed using ABI Prisms Navigator Software. The (+405) G/C and (+936) C/T alleles were observed as different fluorescence peaks in that position.

### Statistical analysis

Snellen VA was converted to the logarithm of minimum angle of resolution (logMAR) VA for the purpose of statistical analysis. Change in VA was calculated as the difference between VA at baseline and VA at follow-up*.*

Statistical calculations were performed using SPSS for Windows 19.0 (SPSS, Chicago, IL, USA). As most data had a skewed distribution, numbers reported are median values unless indicated otherwise and non parametric test methods were used in statistical analyses. Continuous data were analyzed by Mann–Whitney *U*-test and categorical data by Fisher’s exact test. Factors that were significantly associated with s-VEGF after univariate analyses were then entered into multiple regression models to determine the independence of potential correlations and to estimate adjusted ORs (Exp(β)).

Genotype and haplotype analyses were performed with SNP Stats software. Comparisons between genotypes were adjusted using the Bonferroni multiple comparison correction and the reported p-values reflect this correction. The strength of the association between genotypes or alleles in each group was estimated by the calculation of the odds ratios (OR) and 95% confidence intervals (CI). Values of *p* < 0.05 were considered statistically significant.

## Results

### Genotype and allele frequencies

As shown in Table 2, the genotype and allele frequencies of the (+405) CC and (+936) TT were significantly higher in AMD patients than in controls [(OR: 3.86, 95% CI [2.03 - 7.42], *p =* 5 × 10^−6^ and OR: 8.89, 95% CI [1. 05–198.1], *p* = 0.021 respectively)] and [(OR: 1.79, 95%CI [1.3 - 2.48], *p* = 2 × 10^−4^ and OR: 1.95, 95%CI [1. 22–3.12], *p* = 0.003 respectively)]. While, the distribution of (−2578) C/A genotypes and alleles was similar among patients and controls.

**Table 2:** Distribution of genotype and allele frequencies in patients and in controls; in subgroup and in response to anti-VEGF

**SNPs**		**Patients n = 145**	**Controls n = 207**	**G1 n = 117**	**G2 n = 28**	**GR n = 52**	**PR n = 16**
		**n (%)**	**n (%)**	**n (%)**	**n (%)**	**n (%)**	**n (%)**
**(−2578)**	CC	19 (13.10)	38 (18.35)	16 (13.67)	3 (10.71)	4 (7.7)	4 (25)
	CA	67 (46.21)	88 (42.51)	57 (48.71)	10 (35.71)	24 (46.1)	9 (56.2)
	AA	59 (40.69)	81 (39.13)	44 (37.60)	15 (53.57)	24 (46.1)***	3 (18.8)
	C	0.362	0.397	0.380	0.286	0.308	0.531
	A	0.638	0.603	0.620	0.714	0.692	0.468
**(+405)**	GG	55 (37.93)*	97 (46,7)	46 (39.31)	9 (32.14)	15 (28.9)	7 (43.8)
	GC	51 (35.17)	92 (44.4)	42 (35.89)	9 (32.14)	21 (40.4)	6 (37.5)
	CC	39 (26.90)	18 (8.9)	29 (24.78)	10 (35.71)	16 (30.7)	3 (18.8)
	G	0.555*	0.688	0.573	0.482	0.490	0.625
	C	0.445	0.311	0.427	0.518	0.510	0.375
**(+936)**	CC	101 (69.65)	168 (81.1)	83 (70.94)	18 (64.28)	41 (78.8)	12 (75)
	CT	38 (26.21)	38 (18.3)	30 (25.64)	8 (28.57)	11 (21.1)	2 (12.5)
	TT	6 (4.14)**	1 (0.6)	6 (3.41)	2 (7.14)	0	2 (12.5)****
	C	0.828	0.903	0.838	0.786	0.894	0.810
	T	0.172**	0.097	0.179	0.214	0.106	0.190

*Homozygous GG genotype and G allele were significantly higher in AMD patients than in controls [(OR: 3.86, 95%CI [2.03 - 7.42], *p =* 5 × 10^−6^ and OR: 1.79, 95%CI [1.3 - 2.48], *p* = 2 × 10^−4^ respectively)].

**Homozygous TT genotype and T allele were significantly higher in AMD patients than in controls [(OR: 8.89, 95%CI [1. 05–198.1], *p* = 0.021 and OR: 1.95, 95%CI [1. 22–3.12], *p* = 0.003 respectively)].

***Homozygous AA (−2578) genotype was statistically associated with good response [(OR: 0.27, 95%CI [0.07- 1.06], *p* = 0.042)].

****Homozygous TT (+936) genotype was statistically associated with poor response [(OR: 1.61, 95%CI [0.31-8.28], *p* = 0.014].

Otherwise, the distribution of (−2578) C/A, (+405) C/G and (+936) C/T genotypes and alleles frequencies was similar in G1 and G2 patients (Table 1). Subtypes of neovascular AMD did not show statistically significant differences.

### Haplotype analysis

Eight different combined haplotypes of these three commun VEGF SNPs were observed Table 3. The cumulative high risk ACT haplotype was more frequently found in patients with the active form of the disease (G1: 4.6%) compared to those with the scar form (G2: 2.8%), but the difference was not statistically significant (*p* = 0.49).

**Table 3:** Combined haplotypes CA(−2578)/GC(+405)/CT(+936) frequencies in G1 and G2 patients

**Combined haplotypes**	**G1**	**G2**	**OR (95% CI)**	***p *** **value**
**ACC**	0.289	0.428	1.00	**-**
**AGC**	0.242	0.206	0.59 (0.25-1.38)	0.22
**CGC**	0.218	0.131	043 (0.17-1.06	0.069
**CCC**	0.088	0.020	0.13 (0.01-1.12)	0.066
**CGT**	0.070	0.094	1.06 (0.27-4.23)	0.93
**AGT**	0.042	0.051	0.73 (0.15-3.48)	0.7
**ACT**	0.046	0.028	0.48 (0.06-3.85)	0.49
**CCT**	0.004	0.041	2.67 (0.08-93.16)	0.59

### Response to anti-VEGF therapy

Among the 68 G1patients treated with anti-VEGF, at 3 months, 52 patients (85.6%) were classified as good responders and 16 (14.4%) as poor responders.

### VEGF genotyping and response to intravitreal bevacizumab injections

As summarized in Table 2, no difference was shown between the distribution of (+405) G/C genotypes and response to anti-VEGF therapy. However, the patients with genotype homozygous TT (+936) would be poor responders compared with those carrying CT and CC genotypes. Whereas, those with the AA (−2578) genotype would improve their visual outcome compared with AC and CC genotypes *(p* = 0.014; *p* = 0.042 respectively). Nevertheless, theses SNPs lose their significance in analysis of combined haplotypes.

### Determination of VEGF plasma levels

**Figure 1:**
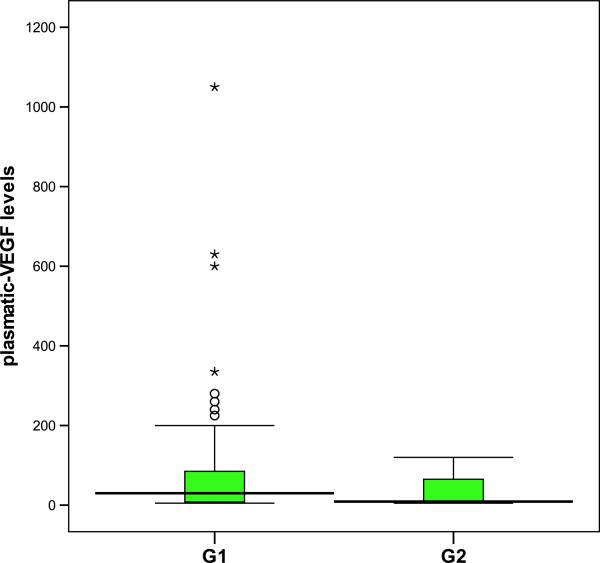
**Distribution of plasma-VEGF levels between the active form of AMD and the scar form.** The mean plasma-VEGF levels was statistically higher in G1 (83,39 pg/ml) compared to G2 (30,60 pg/ml) (p=0.035).

**Figure 2:**
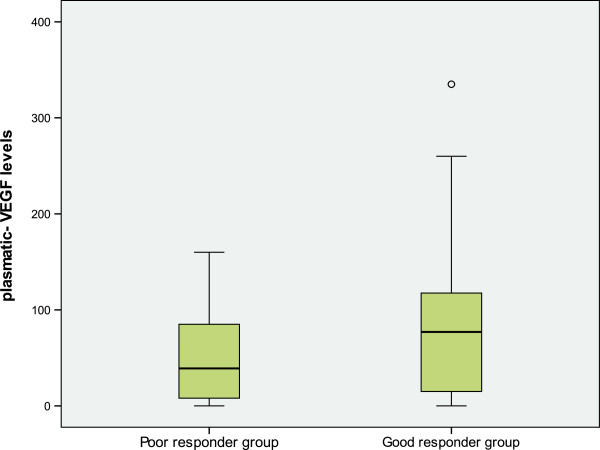
**A box-plot diagram of plasma-VEGF levels stratified by the treatment response groups.** The mean plasma-VEGF levels was higher in GR (86,61 pg/ml) compared to PR (47,12 pg/ml) (p=0.086).

Plasmatic VEGF levels in patients were ranged from 10 to 1050 pg/ml. The mean plasma levels of VEGF was statistically higher in AMD patients (84.22 pg/ml) than in controls (15 pg/ml), and in active form of AMD patients (83.39 pg/ml) compared to those with a scar form (30.60 pg/ml) (*p* = 0.04 and *p* = 0.035, respectively) (Figure 1). However, these results were not confirmed after multivariate analysis and adjustement for known covariates factors (age, gender and risk factors) (Exp (β): 0,95). We did not found any difference in plasmatic VEGF levels between the different types of CNV (*p* = 0.23). Additionally, the mean plasma levels of VEGF in GR patients was higher (86.61 ± 80.30 pg/ml) than in PR patients (47.12 ± 45.74 pg/ml) with a trend to significance *(p = 0.086)* (Figure 2).

### Association of VEGF polymorphisms with serum VEGF levels

If the polymorphism (−2578) C/A showed no relationship with s-VEGF levels of the mutant homozygous genotype (+405) GG and the wild homozygous genotype (+936) CC showed a higher s-VEGF levels than the other genotypes but the differences failed to reach significance.

Additionally, haplotype combinations analysis did not provide any significant association with s-VEGF levels variations.

## Discussion

The current study showed that (+405) G/C and (+936) C/T VEGF polymorphisms were statically associated with exudative AMD in the Tunisian patients corroborating the large study of Haines *et al.*, who attributed to the some SNPs of *VEGF, VLDLR and LRP6 genes*, a role in the risk of AMD development [15]. These results were in close agreement with those previously published by Janik-Papis *et al.*, in a Polish population [16] and by Lin *et al.*, in Taiwan Chinese population [17]. Inversely, it has been reported in other studies that the positive association between VEGF polymorphisms of the gene and susceptibility to the occurrence of AMD could not be confirmed in the Rotterdam study and in Anglo-Celtic subpopulation [18-20]. These controversial results are due to differences in the size of the study, patients heterogeneity, the choice of analyzed SNPs located in the promoter region and/or in the coding regions and the methods used for their genotyping. It is now well established that genetic susceptibility to complex diseases such as AMD, is rather due to the cumulative effect of several predictive alleles identified in the full haplotype information [17,18]. Among combined haplotypes observed in this cohort, the risk ACT variant was more frequently found in patients with the active form of the disease (G1patients) compared to those with the scar form (G2 patients), but the difference was not statistically significant. The small numbers of subjects in G2 could induce a loss of statistical power to detect this difference.

The angiogenesis process is highly controlled through the balance of pro- and anti-angiogenic factors. VEGF is the key pro-factor in this process. Specific polymorphisms in the *VEGF gene* have been associated with a variation of protein levels [7,21,22]. The data from this study support this hypothesis by showing that both in controls and G2 patients compared to the G1 subjects, a significant decrease in s-VEGF was observed, confirming once again that VEGF is the major stimulus for the development and growth of choroidal neovascularization in the exudative AMD because wet AMD involves neovascularization, the best approach for the treatment of CNV appears to be the use of anti-VEGF drugs. At present, several molecules have been used in intravitreal therapies approved by Food and Drug Administration (FDA) and the European Medecines Agency (EMEA).

Among these anti-VEGF agents, several studies report that the bevacizumab (Avastin*; Genentech Inc) gives the best results for the treatment of neovascular AMD [23-26].

The effectiveness of such treatments depends on their molecular weight, the binding affinity of the various isoforms of VEGF and their pharmacokinetics lesional prior retinal barrier. In this study, taking into account of the availability of bevacizumab exposure in plasma, this molecule would be more effective in patients with a high concentration of plasmatic VEGF levels compared to those with the rate of protein relatively low. Indeed, a difference in the distribution of plasma levels of VEGF was found among good responders compared with poor responders. These results corroborate those of Carneiro *et al.*, who show that bevacizumab significantly reduced the plasma levels of VEGF after 28 days after intravitreal injections in patients with exudative AMD compared to injections of ranibizumab, with two main potential determinants of their systemic adverse effects including the blood levels of plasmatic VEGF and the degree of systemic anti-VEGF inhibition.

About the relationship between SNPs of the VEGF gene studied and response to intravitreal injection of bevacizumab, this study revealed, when each SNP was analyzed individually, that the patients mutated homozygous AA (−2578) genotype were rather classified as good responders whereas those with risk homozygous genotype TT(+936) belonged to the group of poor responders. Nevertheless, theses SNPs lose their significance in analysis of combined haplotypes. In the literature, unanimity is not yet fully established for the use of these genetic biomarkers to predict the visual evolution in response to anti-VEGF therapy in the exudative AMD. Indeed, if in the promoter region of VEGF *gene,* the SNP (C-2578A, rs699947) seems to be also, a predictor of success to bevacizumab treatment in Japan population [27], our results, concerning the SNP (C936T, rs3024039) in the 3′Untranslated region, are in discordance with a Qu Y. in Chinese population and Boltz A. studies that provide no evidence for an association between this SNP and the response to bevacizumab treatment [28,29]. In Korean neovascular AMD cohort, Un Chul Park *et al.*, do not find statistically significant effect of VEGF-SNPs (C936T, C-2578A and C-460 T) on visual outcome after ranibizumab treatment [30]. In 5′Untranslate region, the SNP (G405C, rs2010963), despite the fact that it is associated with susceptibility to AMD, the response to bevacizumab was independent of this polymorphisme. This is in agreement with study of Imai *et al.* found that VEGF (−2578; rs699947) and PDEF (+rs113628) were associated with vision changes at 1 month and 3 months post-bevacizumab therapy respectively but VEGF (+405; rs2010963) may not be genetic biomarker to estimate visual outcomes in response to intravitreal bevacizumab treatment for neovascular AMD [27].

In view of these results, the question is what about the genetic profile associated with long-term visual outcome? The follow-up of further study with larger sample sizes and standard intervals to record the results of visual acuity should be performed to confirm these results and to answer this question.

In conclusion, this study found that VEGF genetic variants may contribute to the susceptibility to neovascular AMD in Tunisian patients and were also associated with vision changes at 3 months of anti-VEGF therapy. However, our findings need to be replicated in additional studies. Further expression studies are needed to investigate the potential pharmacologic role of these variants in antiangiogenesis AMD therapy at long-term.

## Competing interests

No benefits in any form have been received or will be received from commercial party related directly or indirectly to the subject of this manuscript.

## Authors’ contributions

IH: carried out the molecular genetic studies and drafted the manuscript. IS: follow up of molecular studies, sequence alignment and statistical analysis. AC: clinical monitoring and identification of patients. FK: clinical monitoring and identification of patients. RB: clinical monitoring and identification of patients. SJ: technical part of the study. MM: technical part of the study. TBA: Director of the research laboratory. LM: clinical department head. YG: follow up of this study, correction of manuscript and corresponding author. All authors read and approved the final manuscript.
